# Sustainable Production of *Ulva* Oligosaccharides via Enzymatic Hydrolysis: A Review on Ulvan Lyase

**DOI:** 10.3390/foods13172820

**Published:** 2024-09-05

**Authors:** Ailan Huang, Xinming Wu, Fuping Lu, Fufeng Liu

**Affiliations:** 1 School of Life Science and Technology, Henan Institute of Science and Technology, Xinxiang 453000, China; 2 College of Biotechnology, Tianjin University of Science & Technology, Tianjin 300457, China; 3 Key Laboratory of Industrial Fermentation Microbiology, Ministry of Education, Tianjin Key Laboratory of Industrial Microbiology, Tianjin 300457, China

**Keywords:** *Ulva* oligosaccharide, bioactive compound, ulvan lyase, *β*-elimination mechanism, structure

## Abstract

Ulvan is a water-soluble sulfated polysaccharide extracted from the green algae cell wall. Compared with polysaccharides, oligosaccharides have drawn increasing attention in various industries due to their enhanced biocompatibility and solubility. Ulvan lyase degrades polysaccharides into low molecular weight oligosaccharides through the *β*-elimination mechanism. The elucidation of the structure, catalytic mechanism, and molecular modification of ulvan lyase will be helpful to obtain high value-added products from marine biomass resources, as well as reduce environmental pollution caused by the eutrophication of green algae. This review summarizes the structure and bioactivity of ulvan, the microbial origin of ulvan lyase, as well as its sequence, three-dimensional structure, and enzymatic mechanism. In addition, the molecular modification of ulvan lyase, prospects and challenges in the application of enzymatic methods to prepare oligosaccharides are also discussed. It provides information for the preparation of bioactive *Ulva* oligosaccharides through enzymatic hydrolysis, the technological bottlenecks, and possible solutions to address these issues within the enzymatic process.

## 1. Introduction

Marine resources are among the most abundant on the planet. In the face of energy shortages and the depletion of non-renewable resources, macroalgae, with its rapid growth and minimal arable land requirements, is considered a promising renewable carbon source [1,2,3,4]. According to their pigments, macroalgae can be classified into brown (Ochrophyta), red (Rhodophyta), and green (Chlorophyta) [5,6]. Green algae has not been well studied compared with red algae and brown algae. Nevertheless, there has been a growing interest and research in green algae since the early 1990s [6]. 

Green algae of the genus *Ulva* grow rapidly in different environments and are the main cause of green tides. Ulvan is a water-soluble sulfated polysaccharide extracted from the cell wall of *Ulva* sp., accounting for about 8% to 29% of the dry weight of the total algae [7,8,9,10]. In addition to ulvan, cell wall polysaccharides include insoluble cellulose, as well as two minor components: alkali-soluble linear xyloglucan and glucuronan [11]. Ulvan has various biological activities, whereas, the application of ulvan is limited due to its high molecular weight, low water solubility, and biocompatibility. In contrast, low molecular weight (LMW) oligosaccharides exhibit improved solubility and biocompatibility, making them more attractive in food and medical fields. Currently, degradation methods include physicochemical approaches such as acid dissolution, ultrasound-assisted extraction, and microwave degradation [12,13]. In comparison, bio-enzymatic hydrolysis has the advantages of mild operating conditions, high efficiency, and substrate specificity [14]. Ulvan lyases (EC number 4.2.2), belonging to the polysaccharide lyase (PL) family, can degrade ulvan into small-molecule oligosaccharides. The cleavage mechanism is similar to other polysaccharide lyase families, cleaving the *β*-1,4 glycosidic bonds by the *β*-elimination mechanism to generate oligosaccharides with unsaturated residues at the non-reducing end [15,16]. 

In recent years, with advancements in genomics and structural biology, there has been a growing number of research characterizing enzymes with ulvan cleavage activity. Furthermore, the determination of ulvan’s lyase structures has significantly enhanced our understanding of catalytic mechanisms. This review aims to highlight the enzymatic production of bioactive *Ulva* oligosaccharides as a more environmentally friendly and efficient approach. The source of ulvan lyases, three-dimensional structures, catalytic mechanisms, as well as preparation methods and industrial applications, are briefly discussed.

## 2. The Biological Activity of Ulvan Polysaccharides and Oligosaccharides 

**Figure 1:**
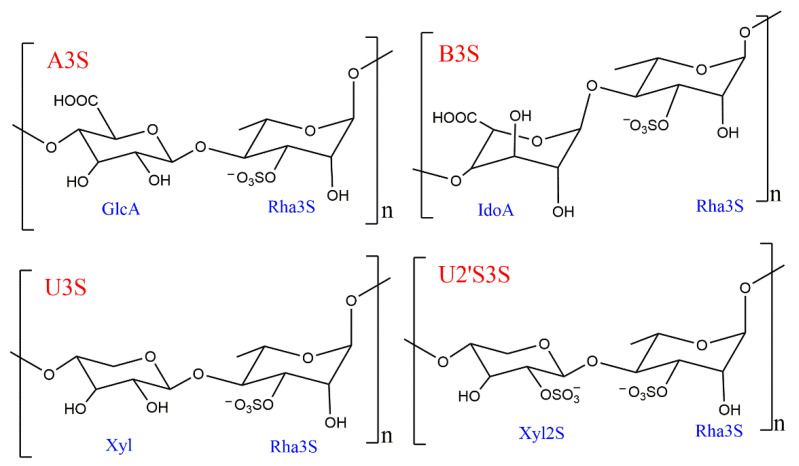
The disaccharide unit structures of ulvan.

The structure of ulvan is mainly composed of sulfated rhamnose (Rha3S), glucuronic acid (GlcA), iduronic acid (IdoA), xylose (Xyl), and xylose-2-sulfate (Xyl2S) [11]. The four disaccharide structural units of the polysaccharide backbone are shown in Figure 1, A3S [→4)-*β*-D-GlcA-(1→4)-*α*-L-3-Rha3S-(1→] and B3S [→4)-*α*-L-IdoA-(1→4)-*α*-L-3-Rha3S-(1→], U3S: [→4)-*β*-D-Xyl-(1→4)-α-L-3-Rha3S-(1→], and U2′S3S: [→4)-*β*-D-sulfated Xyl-(1→4)-*α*-L-3-Rha3S(1→] [6,13]. The content and composition of these disaccharides vary depending on the cultivation environment and extraction method. The structural components, in terms of dry weight percentage, include rhamnose (16.8–45%), xylose (2.1–12%), glucose (0.5–6.4%), glucuronic acid (6.5–19%), sulfate (16–23.2%), and aedugaric acid (1.1–23.2%).

**Figure 2:**
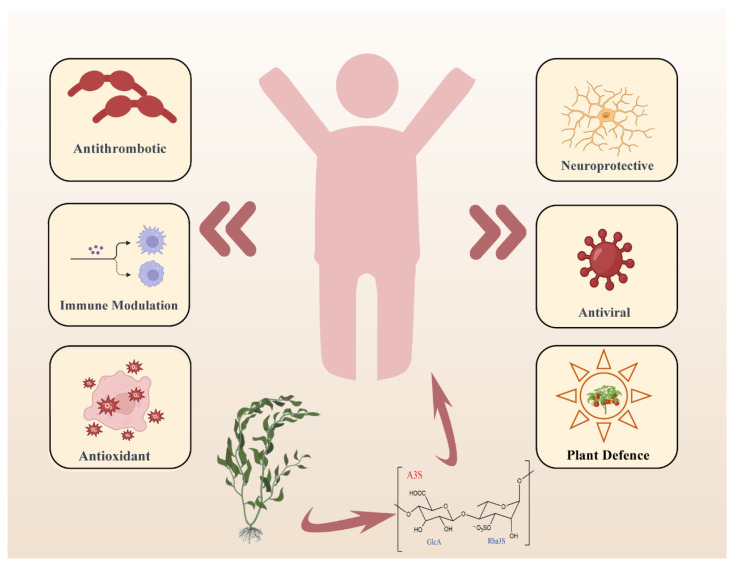
The various physiological activities of ulvan.

Traditionally, ulvan has been a customary dietary component (marine lettuce), valued for its nutritional richness and potential health-enhancing attributes [17,18,19,20,21,22]. However, the high molecular weight and low solubility have limited polysaccharide utilization. In contrast, oligosaccharides possess several advantages due to enhanced solubility and absorption, making them more valuable in the field of functional foods and biomedicine [6,8,13,23,24]. *Ulva* oligosaccharides have various bioactivities (Figure 2), such as antiviral [25,26,27,28], antioxidant activity [27,29,30,31,32,33,34,35], antihyperlipidemic activity [31,36,37,38,39,40], and neuroprotection activity [41,42]. The detailed information of ulvan bioactivities are listed in the Supporting Information (Table S1). 

The ulvan showed the anti-virus bioactivity in vitro or in vivo assay. For example, the ulvan from *Laminaria angustata* algae exhibited inhibitory activity against herpes simplex virus (HSV). It was found that the presence of sulfate groups enhanced the inhibitory ability of polysaccharides against HSV-1 viruses, and the antiviral activity increased with increasing sulfate content [34,43,44]. Chi et al. [28] collected samples of *Ulva pertusa* from the coast of China, which were cleaved by ulvan lyase to generate ulvan oligosaccharide Ulvan-F1. The experimental results showed that, at a concentration of 100 μg/mL, both polysaccharide and its degradation product Ulvan-F1 exhibited significant inhibitory activity against vesicular stomatitis virus (VSV), with inhibition rates of 40.75% and 40.13%, respectively. There is an increasing number of studies on the conformational relationship of the antiviral effects of sulfated polysaccharides, and these data underline the importance of sulfated polysaccharides in the development of antiviral drugs and drug delivery [26,45]. 

The antioxidant capacity of ulvan has been reported. The antioxidant activity of ulvan is mainly attributed to its multiple reactive groups, which trap free radicals, inhibit oxidative reactions, and attenuate oxidative stress-induced cellular damage. Hydroxyl radical scavenging activity is proportional to sulfate content [31]. The sulfated polysaccharides extracted using the enzymatic method contained higher levels of glyoxalates, which enhanced their antioxidant potential. Therefore, oligosaccharides extracted using enzymatic methods are an important source for the development of natural antioxidants and are expected to play an important role in the field of functional foods.

Hyperlipidemia is a common metabolic disorder characterized by abnormally high levels of cholesterol and triglycerides in the blood. Qi and co-workers [36] synthesized high sulfate content ulvan (HU) and evaluated its antihyperlipidemic effects in mice. HU exhibited significantly stronger antihyperlipidemic activity compared to natural ulvan. These results emphasize the importance of sulfate content in enhancing ulvan’s antihyperlipidemic potential. The effects of different molecular weights of ulvan on lipid metabolism [37] and the possible antihyperlipidemic mechanisms of ulvan [38] have been further investigated.

Alzheimer’s disease (AD) is a complex neurodegenerative disorder whose pathogenesis involves multiple mechanisms. One of the proposed mechanisms is the abnormal folding and aggregation of amyloid β-protein (Aβ), which is considered to be one of the major factors in the onset and progression of AD. Recent studies have demonstrated that ulvan can inhibit Aβ aggregation in a concentration-dependent manner [41]. This finding provides a new research direction for the use of ulvan as potential functional foods for the treatment of AD. 

*Ulva lactuca* can be added to the feed of weaned piglets as an alternative source of nutrients and bioactive compounds [46]. Given that green algae contain ulvan, which is indigestible to monogastric animals, the addition of ulvan lyase to the feed could enhance the digestibility of ulvan. The results of experiments showed that the addition of 7% *Ulva lactuca* and 0.01% recombinant ulvan lyase diet to the feed of weaned piglets contributed to an increase in the abundance of intestinal microflora, offering potential applications in the feed industry [47].

Recent studies have reported the role of ulvan and oligosaccharides in plant protection. It has demonstrated that ulvan-treated tomato seedlings and Medicago truncatula exhibit enhanced disease resistance. In tomato seedlings, ulvan treatment led to increased phenylalanine ammonia-lyase (PAL) activity in the leaves [48]. Additionally, the gene expression profiles of ulvan-treated *Medicago truncatula* were similar to those treated with methyl jasmonate [49]. These findings suggest that ulvan has the potential as a bio-elicitor, enhancing the disease resistance of plants.

## 3. Source, Properties, Structures, and Catalytic Mechanism of the Ulvan Lyase

### 3.1. Sources and Categories of Ulvan Lyase

In 1997, Lahaye et al. [50] first detected the activity of ulvan lyase in marine bacteria. With the development of genomics, many ulvan lyase sequences have been included in the Carbohydrate Active enZYmes (CAZy) database (http://www.cazy.org/, accessed on 24 August 2024) [51]. The filamentous fungus *Trichoderma* sp. GL2 produced an extracellular glucuronide lyase (GL) that was active toward ulvan when grown under conditions where glucuronic acid was the sole carbon source [52]. The first isolated ulvan lyase was from the flavobacteria *Persicivirga ulvanivorans*, whose gene encoding the catalytic module had no homologous sequences in the GenBank sequence database and was classified as a new polysaccharide lyase family [53]. This enzyme was identified as an endo-type cleaving the glycosidic bond between glucuronic acid or iduronic acid. Nowadays, the known species that are capable of producing ulvan lyase mainly include *Alteromonas*, *Pseudoalteromonas*, *Formosa agariphila*, and *Nonlabens ulvanivorans.*

Currently, ulvan lyases are divided into five families, that is PL24, PL25, PL28, PL37, and PL40, based on the amino acid sequence. There are seven members of the PL24 family in the CAZy database, which are derived from *Alteromonas* sp. KUL17 [54], *Alteromonas* sp. KUL42 [54], *Alteromonas* sp. LOR_107 [55], *Alteromonas* sp. LOR_61 [55], *Glaciecola* sp. KUL10 [54,56], *Pseudoalteromonas* sp. PLSV_3875 [57] and *Pseudoalteromonas* sp. PLSV_3925 [55]. Two PL25 family members in CAZy are derived from *Alteromonas* sp. A321 [58], and *Pseudoalteromonas* sp. PLSV_3936 [59]. Although LOR_29 [60] from *Alteromonadales* sp. and NLR_492 [60] from *Nonlabens ulvanivorans* were not included in the CAZy database, they have been included in the GenBank under the numbers WP_052010178.1 and WP_036580476.1, respectively. PL28 family members are derived from *Formosa agariphila* KMM 3901 [61], *Nonlabens ulvanivorns* NLR48 [62] and *Nonlabens ulvanivorns* NLR42 [53]. In addition, PL37 [63], as well as PL40 [64], derived from *Formosa agariphila* KMM 3901 [61], were also included in the CAZy database. Besides, Olivier et al. [10] successfully isolated a microbial species capable of secreting ulvan lyase. Through 16S rRNA sequencing, the microbial species’ gene was identified as GIIUL2 and its affiliation with the genus *Alteromonas*. However, in comparison to the registered species available in GenBank, the homology of this species was less than 95%. Consequently, the classification of the ulvan lyase produced by this species into a specific PL family remains uncertain. 

**Figure 3:**
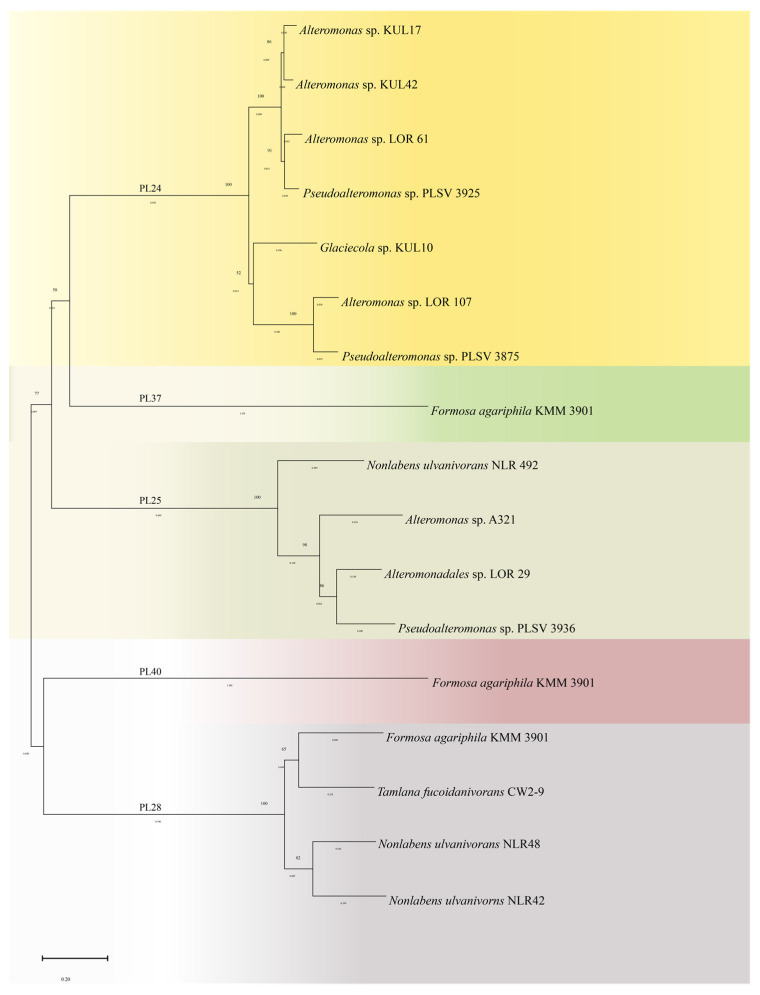
Phylogenetic analysis of ulvan lyase from five families.

A phylogenetic tree was constructed using MEGA 11 [65] software to investigate the relationships among five families of ulvan lyase. As shown in Figure 3, the five families were divided into three main branches on the phylogenetic tree. The PL24 and PL25 families were grouped into one major class, the PL28 family formed another independent branch, and the PL37 and PL40 families clustered on the third branch. This is consistent with the structural information that PL24 and PL25 families have a similar 3D structure, which adopts the *β*-propeller configuration. In contrast, the PL28 family lyase exhibits a *β*-jelly roll structure, suggesting that there are significant structural differences between the enzymes of different families.

### 3.2. Enzymatic Properties of Ulvan Lyase

Temperature and pH are two important factors affecting the enzymatic activity of ulvan lyase. The ulvan lyase exhibits the highest activity within the pH range of 7.5 to 9.0. This is attributed to the fact that most identified ulvan lyases are isolated from the marine bacteria and the intestinal environment of marine organisms, which suggests a potential evolutionary adaptation of marine bacteria to the weak alkaline environment [66].

The optimum temperature for the ulvan lyase enzymatic reaction is 30–50 °C, as shown in Table 1. For example, the optimum temperature of PL24 family *Alteromonas* sp. LOR_107 was 40 °C [55]; for *Pseudoalteromonas* sp. PLSV_3875, this value was 35 °C [57]. Compared with LOR_107, PLSV_3875 has higher stability and still maintained 100% activity after 24 h at 30 °C, while LOR_107 lost 90% activity under the same conditions. Among the PL25 family, *Alteromonadales* sp. LOR_29 had the highest enzymatic activity at 45 °C [60]; the optimum temperature of *Alteromonas* sp. A321 was higher than LOR_29, which was 50 °C [58]. The optimum temperature of ulvan lyase derived from *Formosa agariphila* KMM 3901 of the PL37 family was 40 °C [63]. The *Nonlabens ulvanivorns* NLR42 enzyme exhibited the highest activity at 50 °C. However, this enzyme had poor thermal stability at 50 °C and lost more than 75% of enzymatic activity in 5 min. In contrast, when incubated at 30 °C for 2 h, the enzyme activity did not decrease significantly, which indicated that NLR42 was easily inactivated at high temperatures [53]. 

The enzyme catalytic optimum temperature is higher than the growth temperature of organisms, which is not only found in ulvan lyases. It was reported that many enzymes have optimal catalytic temperatures that are significantly higher or lower than the growth temperature of microorganisms [69]. The differences between microbial optimal growth temperature and enzyme optimal catalytic temperature reflect microorganisms’ adaptive and evolutionary strategies that allow them to maintain physiological function and growth in diverse and fluctuating environments. For instance, the optimal catalytic temperature of PL28 ulvan lyase from *Formosa agariphila* is 45 °C, but it also shows good catalytic activity from 20–50 °C. 

The activity of ulvan lyase was affected by surfactants, metal ions, and chelating agents, and these effects can be divided into activating and inhibitory effects. The enzymatic activity of the PL24 family from *Pseudoalteromonas* sp. PLSV_3875 was activated by Ca^2+^ and Co^2+^, resulting in an increase of 45% and 60%, respectively. The activity was inhibited by Mn^2+^ and Zn^2+^, and the presence of diaminetetraacetic acid (EDTA) also reduced the enzymatic activity [66]. The activity of ALT3695 from *Alteromonas* sp. A321 increased in the presence of Ca^2+^, Mg^2+^ and Ba^2+^. Conversely, low concentrations of Fe^2+^, Cu^2+^, and Co^2+^ resulted in decreased enzyme activity, with complete inactivation occurring at high concentrations. Furthermore, ALT3695 was completely inactivated when exposed to Cd^2+^, Hg^2+^, and Fe^3+^. It is likely attributed to the binding of these ions to the thiol, carboxyl, and amino groups of amino acids, which induces structural alterations and activity loss of the enzyme [58]. Among the PL28 family, the enzyme activity of FaPL28 from *Formosa agariphila* KMM 3901 increased by 70% in the presence of a medium concentration of NaCl (100–200 mM), while the enzyme activity decreased at a higher concentration of NaCl. Meanwhile, the addition of dithiothreitol (DTT) and EDTA decreased the activity by 36% and 63%, respectively. The impact of DTT on enzyme activity implies the potential significance of the four cysteine residues within FaPL28 [61]. These cysteine residues may play a crucial role in either the enzyme’s activity or stability by participating in the formation of disulfide bonds.

### 3.3. The Resolved 3D Structure of Ulvan Lyase

**Figure 4:**
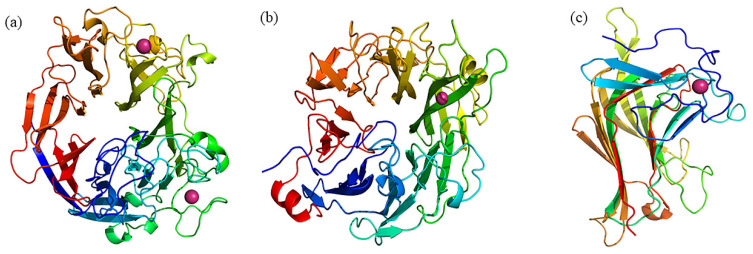
Crystal structures of ulvan lyase of PL24, PL25, and PL28 families. (**a**) LOR_107 of the PL24 family (PDB ID:6BYP) [70], (**b**) PLSV_3936 of the PL25 family (PDB ID: 5UAM) [58], (**c**) NLR48 of the PL28 family (PDB ID:6D3U) [62].

Generally, the enzyme 3D structures are essential for understanding catalytic mechanisms. Three families of ulvan lyase have been resolved, that is LOR_107 of the PL24 family (PDB ID: 6BYP) [70], PLSV_3936 of the PL25 family (PDB ID: 5UAM) [58], and NLR48 of the PL28 family (PDB ID: 6D2C) [62], respectively. Recently, a new structure, Uly1 of the PL24 family (PDB ID: 7CZH) [71], has also been determined, which is consistent with the conformation of 6BYP. The protein structures of three families are shown in Figure 4. The conformation of LOR_107 is consistent with that of PLSV_3936, which both adopt *β*-seven-bladed propeller structure (Figure 4a,b), whereas NLR48 of the PL28 family exhibits a *β*-sandwich jelly-roll conformation (Figure 4c). 

The crystal structure of LOR_107 contains two monomers, and each monomer is folded in the form of a *β-*seven-bladed propeller comprising four antiparallel *β*-strands. The Ca^2+^ metal ions present in the crystal structure are located at the periphery of the propeller, far away from the active site, and play a role in maintaining the stability of the structure.

The PLSV_3936 protein belongs to the PL25 family and also adopts the configuration of *β*-seven-bladed propeller. Among them, blades 1, 2, 3, 4, 5, and 6 consisted of consecutive residues, while blade 7 consisted of four chains, three of which were C-terminal *β*-strands, and the other chain was N-terminal residues. In the PL25 family crystal structure, Zn^2+^ ions are bound between the second and third blades of the propeller, which are closer to the active site. Mutation experiments confirmed that Zn^2+^ ions play a key role in maintaining structural stability, and its deletion will lead to instability in the region near the active site, resulting in the inactivation of the enzyme.

Different from the PL24 and PL25 family’s crystal structures of ulvan lyase, NLR48 of the PL28 family adopts a *β-*sandwich jelly-roll fold structure (Figure 4c), with two concave *β*-folds superimposed on each other. Each *β*-sheet is composed of seven antiparallel *β*-strands. The inner *β*-sheet is composed of *β*1-*β*4-*β*13-*β*6-*β*7-*β*8-*β*9 strands, while the outer *β*-sheet contains *β*2-*β*3-*β*14-*β*5-*β*10-*β*11-*β*12 strands. The inner *β*-sheet forms a deep cleft in which the substrate-binding site resides. The presence of Ca^2+^ ions in the crystal structure provides additional stability to the N-terminus, playing a structural stabilizing rather than catalytic role.

The PL24 family of ulvan lyase has catalytic specificity and only cleaves the glycosidic bond between GlcA-Rha3S. In contrast, the PL25 and PL28 family lyases could cleave the glycosidic bonds between GlcA-Rha3S and IdoA-Rha3S. Ulvan lyases exclusively depolymerize polysaccharides into disaccharide units, yielding a predominant presence of the disaccharide ΔUA-Rha3S in the cleavage products. Presently, ulvan lyases are incapable of cleaving the glycosidic bond between Xyl and Rha3S, resulting in the presence of tetrasaccharides containing Xyl in the enzymatic products.

### 3.4. Catalytic Mechanism of Ulvan Lyase

The biochemical characterization of ulvan lyase indicates its role as an endonuclease cleaving the glycosidic bond between Rha3S and GlcA or IdoA through the *β*-elimination mechanism. The *β*-elimination mechanism is proposed by Gacesa and has been observed across various polysaccharide lyases [59,72,73,74]. This mechanism follows three steps: (1) the neutralization of the negative charge on the carboxyl group in the +1 position of the substrate, thereby lowering the pKa of the C5 proton, (2) abstraction of C5 proton by a base, (3) electron transfer takes place within the carboxyl group and causes the cleavage of the C-O4 glycosidic bond, which creates an unsaturated C4-C5 double bond at the non-reducing end [75]. In general, the residue that neutralizes the negative charge of the +1 subsite of the substrate in the first step is asparagine, aspartic acid, glutamine, glutamic acid, or histidine. According to the nomenclature of Davies et al. for polysaccharides, −1 position represents the non-reducing end, +1 position represents the reducing end, and the cleavage site of the glycosidic bond occurs between the −1 and +1 subsites. 

**Figure 5:**
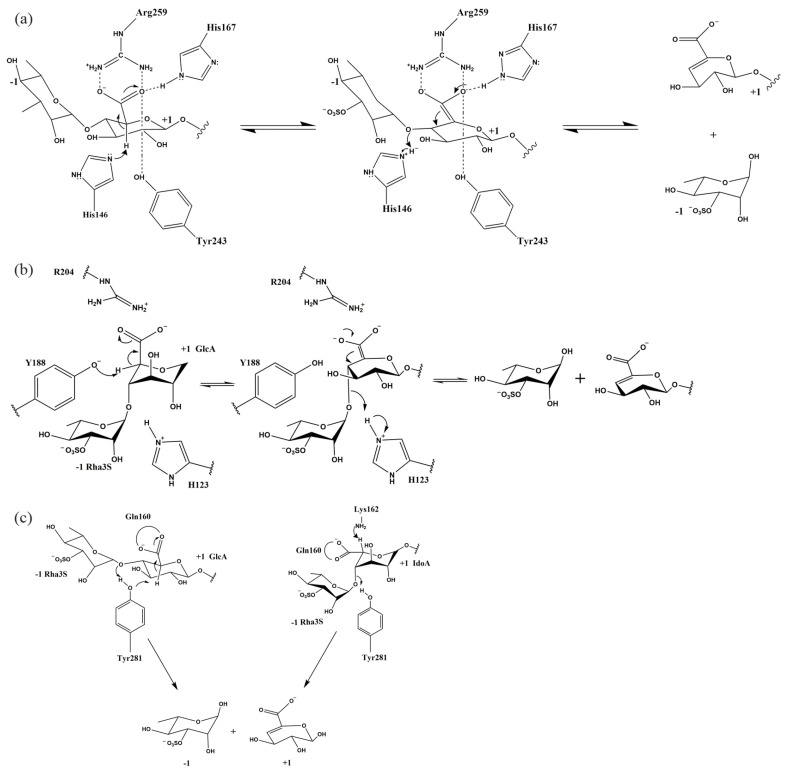
The proposed catalytic mechanisms for ulvan lyase. (**a**) LOR_107 of the PL24 family, (**b**) PLSV_3936 of the PL25 family, (**c**) NLR48 of the PL28 family.

The catalytic mechanisms of the PL24, PL25, and PL28 families have been proposed based on the crystallography analysis and mutation experiments. Within the PL24 family, residues Arg259 and His167 form hydrogen bonds with the GlcA carboxyl group at the +1 position, which neutralizes the negative charge on the carboxyl group of the substrate uronic acid (Figure 5a) [70]. In addition, residue Tyr243 also interacts with the carboxyl group of GlcA. Therefore, these three residues achieve neutralization of the charge on the carboxylate, which reduces the pKa of the C5 proton of +1 position monosaccharide. Residue His146 serves as a base to abstract the C5 proton. On the other hand, His146 also acts as an acid to provide proton, thereby facilitating the formation of double bonds between C4 and C5 and breaking of glycosidic bonds. The mutational experiments provided insights into the distinct roles played by three residues in neutralizing the carboxyl group’s negative charge. Inactivation of the R259A and H167A mutants highlighted the crucial roles of Arg259 and His167 in neutralizing the carboxyl group’s negative charge. The Y243F mutant, while showing residual activity, indicated that residue Tyr243 plays a supportive rather than a primary role in carboxyl group neutralization. Furthermore, the loss of enzymatic activity in the R320N mutant suggests that the arginine mutation at position 320 may cause the Arg259 side chain to deviate towards the main chain Gly262-Asn263. This deviation likely triggers subtle rearrangement in the protein structure, which can result in Arg259 being unable to neutralize the acidic group of GlcA in the substrate and hindering the extraction of the C5 proton. This indirectly suggests the importance of Arg259 in charge neutralization. 

The crystallographic analysis of the enzyme complexed with a tetrasaccharide substrate containing GlcA, in combination with mutagenesis experiments, was undertaken to identify key residues responsible for catalyzing the cleavage of the GlcA to Rha3S glycosidic bond within the PL25 family. As depicted in Figure 5b [59], the complex structure unveiled the role of Arg204 in neutralizing the acidic group of the aronic acid in the +1 position, with help from His143 and Tyr246. Residue Tyr188 is located near the C5 proton at the +1 position and most likely acts as a general base to the abstract C5 proton. His123, as an acid, will transfer the proton to the bridging oxygen. Breakage of the C4-O4 bond and formation of the C4-C5 double bond will then occur. However, the complex structure of the ΔUA-R3S-IdoA-R3S substrate with the enzyme is currently unresolved, leaving the mechanism for cleavage of the IdoA-containing polysaccharide glycosidic bond not fully elucidated. Despite this, mutation experiments showed that mutations in Arg204, His123, and Tyr188 resulted in enzyme inactivation, strongly suggesting that these residues play an important role in catalysis.

As shown in Figure 5c [62], based on the resolved crystal structure and alignment with other polysaccharide lyases, the mechanism of the PL28 family was deposited. The PL28 targets different cleavage residues containing GlcA and IdoA glycosidic bonds. It is proposed that Gln160 attenuates the pKa of the acidic group by forming two hydrogen bonds with the carboxyl group of GlcA in the +1 position. Arg117 residue also forms a hydrogen bond with the carboxyl group of GlcA, which can further neutralize the negative charge in the carboxyl group of GlcA in the +1 position. From the position of the residues in the crystal structure, the hydroxyl group of Tyr281 points to the proton and could act as a base to abstract the C5 proton. At the same time, this residue acts as an acid, providing a proton to the bridging oxygen, prompting the breaking of the C4-O glycosidic bond. In the IdoA-containing glycosidic linkage case, Tyr281 is located on the other side of the C5 proton and is, therefore, unlikely to extract the proton as a base. Therefore, the hydroxyl group of Lys162 is thought to abstract the proton at the C5 position based on structural alignment with the PL7 and PL13 families. The residues involved in catalysis in the three families are listed in Table 2.

## 4. Molecular Modification of Ulvan Lyase 

Enzyme stability is a critical factor in the industrial production of oligosaccharides. Ulvan lyases from natural resources showed low stability. Therefore, molecular modification of the enzyme is required to improve its stability and activity. The identification of key residues involved in catalysis is essential for enzyme modification. Ulaganathan et al. [59] found that amino acid residues His123, Tyr188, Arg204, and His264 play a key role in substrate catalysis of PLSV_3936. In addition, Qin et al. [66] used a similar approach to construct mutants of ASPL from the genus *Alteromonas*; it was indicated that mutants H146A, H167A, R259A, R320A, N263F, Y243F, and H384A lost their activity, indicating the importance of these residues in substrate catalysis.

**Figure 6:**
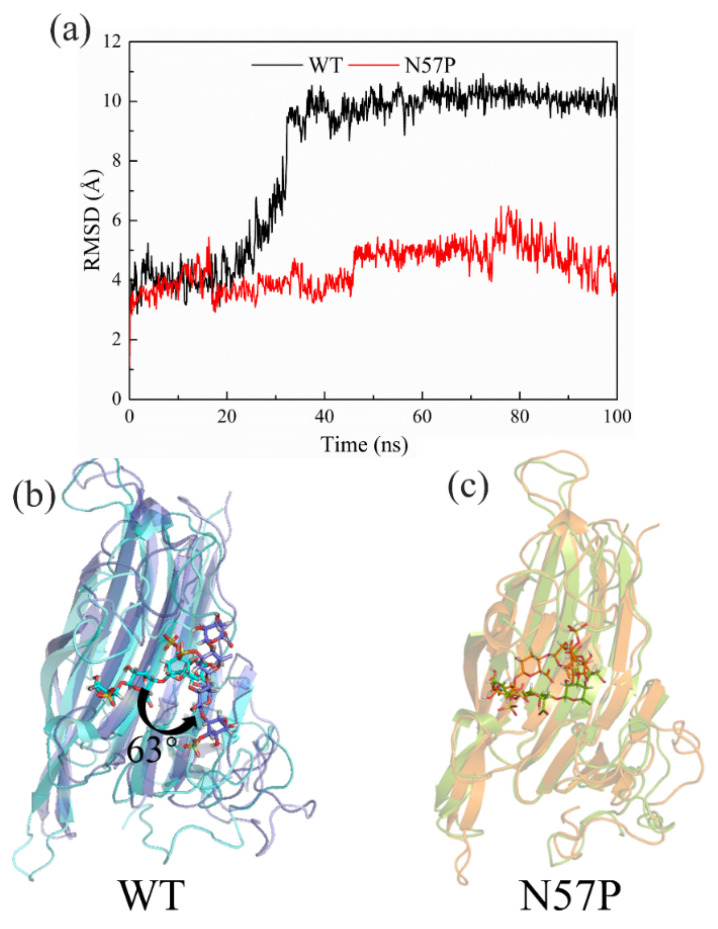
The substrate along with the 100 ns MD simulations of WT and mutant N57P. (**a**) The RMSD of the substrate in WT and N57P from the MD simulations; (**b**) the protein and substrate structures of WT at 0 ns and 100 ns, the complex structure of 0 ns was shown in cyan, structure of 100 ns was shown in purple; (**c**) the protein and substrate structures of N57P at 0 ns and 100 ns, the structure of 0 ns was shown in orange, structure of 100 ns was shown in pale green.

Relative to molecular modification and protein engineering studies of brown algae of alginate lyase [76,77], there is less research on ulvan lyase. Several methods, including disulfide bond engineering [78] and truncation of non-catalytic domains [79,80], have been employed to improve the thermal stability of alginate lyase. An engineered N57P mutant of ulvan lyase from Nonlabens ulvanivorans was recently rationally designed [81]. This mutant demonstrates significantly improved catalytic efficiency and thermostability compared to the wild type (WT). Molecular dynamics (MD) simulations provided insights that N57P reduces protein flexibility through the proline substitution in the flexible loop region E47-N57. This reduction in flexibility indirectly contributed to the stability of the adjacent loop regions, specifically T102-R117 and G209-R216. Consequently, the substrate anchored in the active site, resulting in the enhanced catalytic efficiency observed in N57P. The root-mean-square deviation (RMSD) of the substrate and changes of position from 0 ns to 100 ns in WT and N57P are shown in Figure 6. Nevertheless, the investigation of ulvan lyase protein engineering is still limited; more studies are needed to improve the stability and enzymatic activity. Based on the N57P mutant, N57P/Q229M was further iterated to obtain N57P/Q229M (both enzyme activity and thermal stability were enhanced). MD results showed that the N57P/Q229M mutation improves thermal stability by introducing additional hydrogen bonds [82].

## 5. Production of Ulvan Lyase

The hydrolysis conditions of different families of enzymes are different, but the hydrolysis pH is weakly alkaline, and the temperature is mostly in the range of 30–50 °C. For instance, 5 mg sulfated polysaccharides extracted from *Ulva lactuca* were dissolved in 0.5 mL of 20 mM Tris buffer (pH 8.5) containing 150 mM NaCl. After adding 50 μg enzyme, the mixture was incubated at 37 °C for 12 h to obtain the end products [83]. These products were separated by size-exclusion chromatography, yielding oligosaccharides of different molecular structures. Currently, the oligosaccharides obtained from enzymatic hydrolysis of ulvan are complex mixtures of various molecular weights rather than single molecules.

Further structural analysis of the oligosaccharide fragments using 1H NMR and LC-MS revealed that the fragments included disaccharides, trisaccharides, tetrasaccharides, and pentasaccharides. The disaccharide structure consisted of unsaturated uronic acid (ΔUA) linked to Rha3S (ΔUA-Rha3S), the trisaccharide structure was Rha3S-Xyl-Rha3S, the tetrasaccharide had an unsaturated uronic acid at the non-reducing end (ΔUA-Rha3S-Xyl-Rha3S), and the pentasaccharide was Rha3S-Xyl-Rha3S-Xyl-Rha3S, along with some branched oligosaccharides. Experimental results showed that as the enzymatic reaction time increased, the detected oligosaccharide chain length gradually decreased, but only disaccharide units were obtained in the end, with no monosaccharides detected.

Additionally, Qiao et al. [84] explored fermentation conditions in 5 L and 30 L bioreactors and optimized the fermentation process using kinetic models. The optimal conditions were found to be 28–32 °C and an initial pH of 7.0, demonstrating the potential for industrial-scale production of ulvan lyase. After 20 h of fermentation, the enzyme activity reached 1.20 U/mL, which further validated the feasibility of industrial-scale production of ulvan lyase.

The composition of the medium and the culture conditions of strain are the key factors affecting the expression and enzyme activity of ulvan lyase. Luria-Bertani medium with peptone and yeast powder was utilized in most fermentation processes [58,66]. In addition, other researchers utilized ZoBell medium containing aged seawater as fermentation conditions [53]. 

## 6. Conclusions and Prospects

*Ulva* oligosaccharides possess a wide range of biological activities, so they have huge potential for use in food, medicine, and cosmetics. Ulvan lyases are the most effective polysaccharide lyases for the bioconversion of polysaccharides into oligosaccharides, and the production of oligosaccharides using this enzyme is undoubtedly a sustainable and environmental-friendly method. Extensive research has been carried out on the evolutionary relationships between crystal structures and catalytic mechanisms of different families of ulvan lyases. However, compared to polysaccharide lytic enzymes from red and brown algae, such as alginate lyases and carrageenases, ulvan lyase still requires more exploration and research.

The structure of protein is crucial to understanding the catalytic mechanism of enzymes. Therefore, the more high-resolution resolved protein structure will enhance the understanding of structure–function relationships. In addition to structural biology, computational techniques such as Alphafold2, can be used for predicting protein structures. MD simulation and quantum mechanics/molecular mechanics (QM/MM) tools could also be used to guide the design and modification of enzymes to improve their catalytic properties, including activity, substrate specificity, and thermal and pH stability. Specifically, in light of the distinctive catalytic proficiency exhibited by PL24 family lyase, it demonstrates the capability to selectively generate IdoA tetrasaccharides. The PL25 and PL28 family lyases have the potential for large-scale preparation of disaccharides. In addition to improving the catalytic performance of the ulvan lyase, the benefits of enzymatic hydrolysis can be achieved by screening high enzyme-producing strains and genetic engineering of strains to enable them to survive under industrial production conditions. These improvements will provide more possibilities for the industrial enzymatic production of oligosaccharides and marine-derived functional foods and applications in various fields.

## Figures and Tables

**Table 1:** The information of the characterized ulvan lyase in five families.

Family	GeneBank	Bacteria	T_opt_/°C	Optimal pH	MW/kDa ^1^	Ref.
PL24	BAY00694.1	*Alteromonas* sp. KUL17	-	-	55	[54]
	BAY00695.1	*Alteromonas* sp. KUL42	-	-	111.23	[54]
	AMA19991.1	*Alteromonas* sp. LOR_107	40	8.0	59.64	[55,66]
	WP_032096165.1	*Alteromonas* sp. LOR_61	-	-	110.9	[55]
	BAY00693.1	*Glaciecola* sp. KUL10	-	-	112.74	[54,56]
	AMA19992.1	*Pseudoalteromonas* sp. PLSV_3875	35	8.0	59	[55,57]
	WP_033186955.1	*Pseudoalteromonas* sp. PLSV_3925	-	-	111.4	[55]
PL25	QFR04505.1	*Alteromonas* sp. A321	50	8.0	53	[58]
	WP_033186995.1	*Pseudoalteromonas* sp. PLSV_3936	-	-	54.28	[59]
	WP_052010178.1	*Alteromonadales* sp. LOR_29	45	7.5	52	[60]
	WP_036580476.1	*Nonlabens ulvanivorans* NLR_492	-	-	55	[60]
	WP_129740764.1	*Alteromonas* sp. *76–1*	45	9.0		
PL28	CDF79931.1	*Formosa agariphila* KMM 3901	45	8.5	54.73	[61]
	KEZ94336.1	*Nonlabens ulvanivorans* NLR48	-	-	30	[62]
	AEN28574.1	*Nonlabens ulvanivorns* NLR42	50	9.0	46	[53]
	WP_139698591	*Tamlana fucoidanivorans* CW2-9	40	9.0	52	[67]
PL37	CDF79930.1	*Formosa agariphila* KMM 3901	40	8.0	69.31	[63]
PL40	CDF79911.1	*Formosa agariphila* KMM 3901	35	8.0	30	[68]
Uncategorized		*Alteromonas* sp. GIIUL2	35	8.0		[10]

^1^ MW: molecular weight.

**Table 2:** Residues participated in catalysis in three families of ulvan lyase.

Enzymes	Monosaccharides Contained in the Substrate	Catalytic Base	Catalytic Acid	Neutralization of Negative Charge in the Substrate
PL24	GlcA	His146	His146	Arg259 His167 Tyr243
PL25	GlcA	Tyr188	His123	Arg204
PL28	GlcA	Tyr281	Tyr281	Gln160 Arg117
PL28	IdoA	Lys162	Tyr281	Gln160 Arg117

## Data Availability

No new data were created or analyzed in this study. Data sharing is not applicable to this article.

## Supplementary Materials

The following supporting information can be downloaded at: https://www.mdpi.com/article/10.3390/foods13172820/s1, Table S1: The bioactivities of ulvan and detailed information. Reference [85] is cited in the Supplementary Materials.
