# Supplementary material for: Sustainable Production of Ulva Oligosaccharides via Enzymatic Hydrolysis: A Review on Ulvan Lyase
Source: Foods. 2024 Sep 5;13(17):2820. doi: 10.3390/foods13172820 (PMC11395424; doi:10.3390/foods13172820)
Supplement: Supplementary file 1 [file foods-13-02820-s001.zip › foods-3071969-supplementary.pdf]

# Sustainable Production of *Ulva* Oligosaccharides *via* enzymatic hydrolysis: A review on Ulvan Lyase

**Ailan Huang [1,2], Xinming Wu [2], Yang Wang [2], Fuping Lu [2,3], Tianyou Yang [1,*] and Fufeng Liu [2,3,*]**

[1] School of Life Science and Technology, Henan Institute of Science and Technology, Xinxiang 453000, China
[2] College of Biotechnology, Tianjin University of Science & Technology, Tianjin 300457, China
[3] Key Laboratory of Industrial Fermentation Microbiology, Ministry of Education, Tianjin Key Laboratory of Industrial Microbiology, Tianjin 300457, China
* Correspondence: yangtianyou2004@163.com (T.Y.); fufengliu@tust.edu.cn (F.L.);
Tel.: +86-13837345613 (T.Y.); +86-22-60602717 (F.L.); Fax: +86-22-60602298 (F.L.)

**Table S1.** The bioactivities of ulvan and detailed information

| Bio-activity | Species | Detail information | Reference |
|---|---|---|---|
| Antiviral | *Laminaria angustata* | Inhibition of herpes simplex virus | [1] |
| | *Ulva pertusa* | Inhibition of vesicular stomatitis virus | [2] |
| Antioxidant | *Ulva pertusa* | Low molecular weight and high sulfate content are more effective | [3] |
| Antihyperlipidemic | *Ulva pertusa* | Low molecular weight polysaccharide are more effective | [4] |
| | *Ulva pertusa* | Polysaccharides with high sulfate content are more effective | [5] |
| Neuroprotective | — | Attenuate $\alpha$-syn aggregation *in vitro* | [6] |
| Immunomodulators for Animal Feeding | *U. lactuca* | Enhance animal disease resistance | [7] |
| Plant Defence | *U. lactuca* | Increase disease resistance in tomato seedlings | [8] |
| | *U.* | Increase disease resistance | [9] |

[1] Saha, S.; Navid, M. H.; Bandyopadhyay, S. S.; Schnitzler, P.; Ray, B., Sulfated polysaccharides from *Laminaria angustata:* Structural features and in vitro antiviral activities. *Carbohydr. Polym.* **2012,** *87* (1), 123-130.

[2] Chi, Y.; Zhang, M.; Wang, X.; Fu, X.; Guan, H.; Wang, P., Ulvan lyase assisted structural characterization of ulvan from *Ulva pertusa* and its antiviral activity against vesicular stomatitis virus. *Int. J. Biol. Macromol.* **2020,** *157*, 75-82.

[3] Coste, O.; Malta, E. J.; López, J. C.; Fernández-Díaz, C., Production of sulfated oligosaccharides from the seaweed *Ulva* sp using a new ulvan-degrading enzymatic bacterial crude extract. *Algal Res.* **2015,** *10*, 224-231.

[4] Pengzhan, Y.; Ning, L.; Xiguang, L.; Gefei, Z.; Quanbin, Z.; Pengcheng, L., Antihyperlipidemic effects of different molecular weight sulfated polysaccharides from *Ulva pertusa* (Chlorophyta). *Pharmacol. Res.* **2003,** *48* (6), 543-549.

[5] Qi, H. M.; Huang, L. Y.; Liu, X. L.; Liu, D. M.; Zhang, Q. B.; Liu, S. M., Antihyperlipidemic activity of high sulfate content derivative of polysaccharide extracted from *Ulva pertusa* (Chlorophyta). *Carbohydr. Polym.* **2012,** *87* (2), 1637-1640.

[6] Wang, W.; Wang, X.; Gao, W.; Cui, Z.; Zhang, H.; Lu, F.; Liu, F., Ulvan inhibits alpha-synuclein fibrillation and disrupts the mature fibrils: In vitro and in vivo studies. *Int. J. Biol. Macromol.* **2022,** *211*, 580-591.

[7] Ribeiro, D. M.; Costa, M. M.; Trevisi, P.; Carvalho, D. F. P.; Correa, F.; Martins, C. F.; Pinho, M.; Mourato, M.; de Almeida, A. M.; Freire, J. P. B.; Mestre Prates, J. A., Piglets performance, nutrient digestibility and gut health in response to feeding *Ulva lactuca* seaweed supplemented with a recombinant ulvan lyase or a commercial carbohydrase mixture. *J. Anim. Physiol. Anim. Nutr. (Berl.)* **2024**, 17.

[8] El Modafar, C.; Elgadda, M.; El Boutachfaiti, R.; Abouraicha, E.; Zehhar, N.; Petit, E.; El Alaoui-Talibi, Z.; Courtois, B.; Courtois, J., Induction of natural defence accompanied by salicylic acid-dependant systemic acquired resistance in tomato seedlings in response to bioelicitors isolated from green algae. *Sci. Hortic.* **2012,** *138*, 55-63.

[9] Jaulneau, V.; Lafitte, C.; Jacquet, C.; Fournier, S.; Salamagne, S.; Briand, X.; Esquerre-Tugaye, M. T.; Dumas, B., Ulvan, a sulfated polysaccharide from green algae, activates plant immunity through the jasmonic acid signaling pathway. *J Biomed Biotechnol* **2010,** *2010*, 525291.
